# Dr. James M. Jenkins

**Published:** 1917-03

**Authors:** 


**Dr. James M. Jenkins,** Syracuse 1875, died at his home in Auburn Feb. 3, aged 67.

